# One Step e-Beam Radiation Cross-Linking of Quaternary Hydrogels Dressings Based on Chitosan-Poly(Vinyl-Pyrrolidone)-Poly(Ethylene Glycol)-Poly(Acrylic Acid)

**DOI:** 10.3390/ijms21239236

**Published:** 2020-12-03

**Authors:** Ion Călina, Maria Demeter, Anca Scărișoreanu, Veronica Sătulu, Bogdana Mitu

**Affiliations:** 1 National Institute for Lasers Plasma and Radiation Physics, 409 Atomiștilor, 077125 Măgurele, Romania; calina.cosmin@inflpr.ro (I.C.); anca.scarisoreanu@inflpr.ro (A.S.); veronica.satulu@inflpr.ro (V.S.); 2 Faculty of Physics, University of Bucharest, 405 Atomiștilor, 077125 Măgurele, Romania

**Keywords:** hydrogel, e-beam cross-linking, swelling, ibuprofen, network parameters

## Abstract

We report on the successful preparation of wet dressings hydrogels based on Chitosan-Poly(N-Vinyl-Pyrrolidone)-Poly(ethylene glycol)-Poly(acrylic acid) and Poly(ethylene oxide) by e-beam cross-linking in weakly acidic media, to be used for rapid healing and pain release of infected skin wounds. The structure and compositions of hydrogels investigated according to sol-gel and swelling studies, network parameters, as well as FTIR and XPS analyses showed the efficient interaction of the hydrogel components upon irradiation, maintaining the bonding environment while the cross-linking degree increasing with the irradiation dose and the formation of a structure with the mesh size in the range 11–67 nm. Hydrogels with gel fraction above 85% and the best swelling properties in different pH solutions were obtained for hydrogels produced with 15 kGy. The hydrogels are stable in the simulated physiological condition of an infected wound and show appropriate moisture retention capability and the water vapor transmission rate up to 272.67 g m^−2^ day^−1^, to ensure fast healing. The hydrogels proved to have a significant loading capacity of ibuprofen (IBU), being able to incorporate a therapeutic dose for the treatment of severe pains. Simultaneously, IBU was released up to 25% in the first 2h, having a release maximum after 8 h.

## 1. Introduction

A wound is defined as a fault or a break of the skin caused by physical, chemical, and thermal injury [1]. Subsequent wound infections and inflammation caused by various bacterial strains often lead to challenging complications for both personal and public health during wound care management [2]. The dressing is an important component contributing to wound healing in the shortest possible time and to protection against bacterial infection, with minimal pain and discomfort to the patients, leading to tissue repair and regeneration [3,4].

Hydrogel based dressings prepared from natural and synthetic biocompatible polymers can prevent microbial infection, absorb large wound exudates, and can be used as vehicles for the simultaneous release of various anti-inflammatory or analgesic drugs to the wound site. Besides, hydrogel wound dressings will not stick to the wound, offering further benefits [5]. Hydrogels are 3D cross-linked networks having high water retention capacity obtained through various techniques, including radiation cross-linking [6,7], freeze-thawing cycles [8,9], and chemical synthesis [10].

The radiation cross-linking is a well-established technique for developing sterile hydrogel wound dressings. The e-beam synthesis of the hydrogel gives homogeneity in the hydrogel network structure, while the degree of cross-linking, which strongly determines the extent of swelling, can be easily controlled by varying the absorbed dose [11,12].

Similar to other radiation technologies, the e-beam radiation initiates chemical reactions in dilute or semi-dilute aqueous solutions which give rise to a series of reactive species as the main results of the water radiolysis. Of these, radical species and molecular products as $e_{aq}^{-}$, HO•, H•, H_2_, H_2_O_2_, and H_3_O^+^, with different reactivities are produced. In deoxygenated or Ar-saturated polymers solution, the hydroxyl radical (HO•) and $e_{aq}^{-}$ presents the highest chemical yields [13]. The HO• and H• radicals react rapidly with polymer chains through hydrogen abstraction which produces several polymer macroradicals depending on the initial concentration of the polymers. The resulted macroradicals participate in intra-and intermolecular free-radical recombination reactions, finally forming a crosslinked polymeric networks with stable and permanent structure [14].

Several natural and synthetic polymers can be used for biomedical hydrogels production designed for wound treatments. Recently, biopolymers based wound dressings have been widely used, among them being chitin/chitosan [15,16,17], collagen [18], cellulose [19], and gelatin [20] due to their biocompatibility, biodegradability, nontoxic, analgesic, and moisture retention capacity. Chitosan (CS) is a derivative of chitin (poly-*N* acetyl glucosamine), which is the second-most abundant biopolymer after cellulose [21]. CS and its derivatives have been widely used as a natural source for hydrogels in many fields, including wound dressing [22,23], tissue engineering [24], drug delivery applications [25], and gene delivery [26]. Poly(*N-*vinyl pyrrolidone) (PVP) is a linear synthetic polymer, is non-toxic and biocompatible, and it has been used as the main component of temporary skin covers or wound dressing [27].

The miscibility of chitosan and PVP has been reported as a result of the interaction between the C = O groups of the pyrrolidone rings of PVP and the amino and OH groups of CS, by forming H-bonds and thus producing material with novel characteristic [28]. Gamma-irradiation of CS-PVP pH-sensitive hydrogels was reported by Dergunov et al. [29]. In that study, the adsorption of bovine serum albumin in CS-PVP hydrogels and their release behavior have been investigated and it has been found that the adsorption capacity of hydrogels increases with increasing CS content in the gel system. Risbud et al. developed a pH-sensitive CS-PVP hydrogel for the controlled release of antibiotics in the gastric environment [30].

The interaction of CS, PVP, and polyethylene glycol (PEG) has been reported by several groups in the last decades. Mahmud et al. reported the characterization of hydrogels produced from CS and PVP via γ-radiation and evaluated the effect of PEG on hydrogels in terms of cross-linking density, gel fraction, swelling degree, syneresis effect at a different temperature, and morphological structure [31]. Also, Das et al. [32] showed that the presence of PEG in CS-PVP hydrogels increases the release rate of active ingredients such as drugs embedded in the hydrogel networks. Li et al. investigated the physical and functional properties of CS films prepared with different content of PVP and poly(ethylene oxide) (PEO) and showed that the blending of CS-PVP does not affect the physical and chemical properties of CS significantly [33]. Rasool et al. prepared CS-PVP-Poly(acrylic acid) (PAA) for the controlled release of Ag-sulfadiazine and demonstrated that such polymeric systems can successfully be used as wound healing and wound dressing for drug delivery [34].

Mozalewska et al. [35] developed a hydrogel wound dressing based on chitosan-PVP-agar by radiation-initiated cross-linking. The studies showed antimicrobial character of the CS-based hydrogel towards Gram-positive bacterial strain. Zhao et al. prepared hydrogels using carboxymethylated chitosan (CM-chitosan) by e-beam irradiation at room temperature and studied the mechanical properties, gel fraction, and swelling behavior [36].

The present work reports on the preparation and evaluation of CS-PVP-PEG-PAA or CS-PVP-PEO-Poly(lactic acid) (PAL) hydrogels via e-beam cross-linking. To the best of our knowledge, no study has been carried out on such hydrogel quaternary system to be used as a wet dressing for rapid healing and pain release of infected skin wounds and at the same time to be used as release vehicles for anti-inflammatory drugs as is ibuprofen.

The hydrogel properties were evaluated following sol-gel analysis, swelling studies, moisture retention capability, water vapor transmission rate, and network parameters, as well as FTIR and XPS analyses showed the efficient interaction of the hydrogel components upon irradiation. Further, the synthesized hydrogel was explored for its encapsulation efficiency, drug loading capacity and in vitro release of ibuprofen (IBU). These novel hydrogels showed potential for use in biomedical applications, particularly as wound healing dressings.

## 2. Results and Discussion

### 2.1. The Reaction Mechanism of CS-PVP-PEG-PAA Hydrogels

**Scheme 1:**
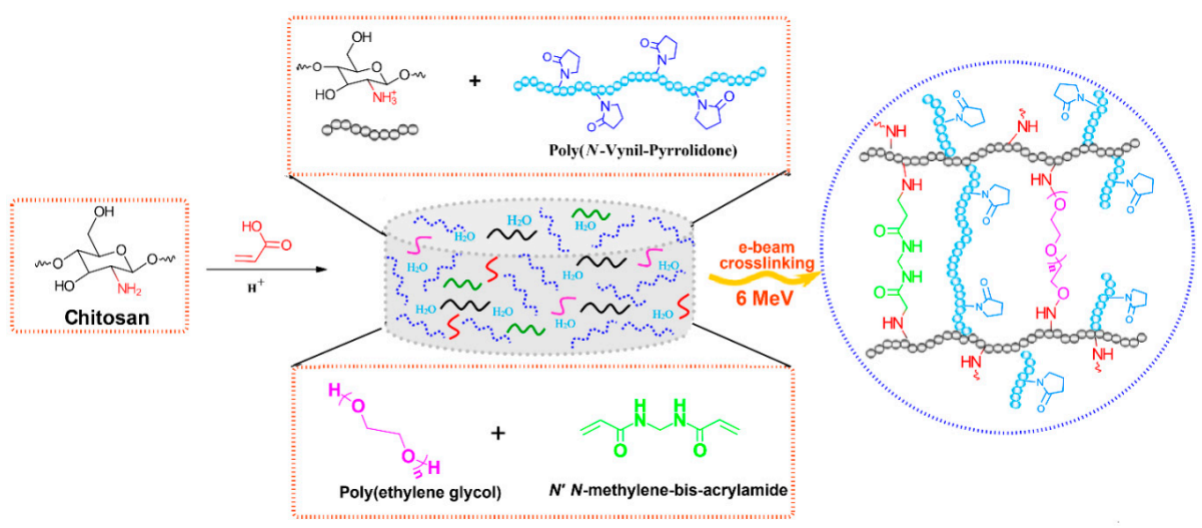
Schematic illustration of the preparation CS-PVP-PEG-PAA hydrogels via e-beam cross-linking and the proposed crosslinked structure.

In Scheme 1 it is presented the reaction mechanism of e-beam radiation cross-linking of CS-PVP-PEG-PAA hydrogels. High absorbed dose and dose rate generated throughout e-beam irradiation process of complex aqueous polymers solutions lead to the formation of considerable amounts of macroradicals which subsequently participate in radical recombination reactions. As a result of the recombination reactions, new crosslinked bonds within polymers macroradicals are generated, forming a new and more stabilized and compact hydrogel structure. The reaction mechanism describes the structures of substrates and the resulted structure of the e-beam crosslinked hydrogel.

### 2.2. Sol-Gel Analysis

**Figure 1:**
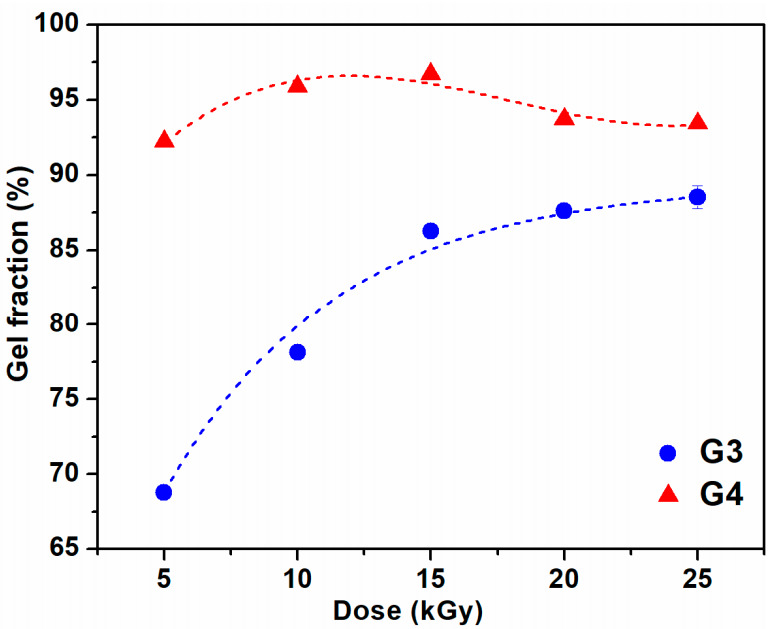
Gel fraction as a function of irradiation dose for G3 and G4 hydrogels.

The variation of gel fraction of G3 and G4 hydrogels synthesized by e-beam cross-linking at various doses is illustrated in Figure 1. The result indicates that the gel fraction of G3 hydrogels constantly increased with absorbed dose and reaches a maximum value of 87% at the dose of 25 kGy. For this composition, lower doses below 15 kGy lead to a hydrogel with reduced gel content, below 85%. In contrast, for the G4 composition even at doses below 15 kGy, the gel content reached above 90%, having a maximum of 96% at 15 kGy. Above 15 kGy, the G4 hydrogels present a slight decrease of the gel fraction which could be correlated with the degradation of the hydrogel structure which becomes more dominant than cross-linking at high doses. Previous studies on CS-PVP hydrogels reported a gel fraction of about 80% for hydrogels prepared from chitosan and PEG diacrylate (PEGDA) [37] and PVP-CM-chitosan hydrogels [38].

In our case, the ${\;p}_{0}{/q}_{0}$ has a value of 0.28 for G3 hydrogel, which suggests that cross-linking processes are predominant. The higher ${\;p}_{0}{/q}_{0}$ value of 0.51 obtained for G4 hydrogel is indicating, besides the above-mentioned cross-linking processes, a moderate contribution of chain scission processes. The ${\;p}_{0}{/q}_{0}$ ratios, $D_{g}$ and $D_{v}$ are presented in Table 1. Similar values were obtained for hydrogel dressings based on PVP/chitosan/agar [35] and PVP/PAA [39].

Other significant parameters characterizing the changes induced by ionizing radiation in the polymer are the radiation yield of the cross-linking, G(X), which provides information on the amount of new bonds formed per unit of absorbed energy, and respectively the radiation yield of chain scission, G(S), which counts the number of broken bonds in the backbone of the polymer per unit of absorbed dose. Both processes occur simultaneously and their yields determine the final results of irradiation [40]. The G(X) and G(S) (mol/J) values can be calculated with the following equations:

(1) $$G\left(X\right)=\frac{4.9\cdot 10^{2}\cdot c}{M_{C}\cdot D\cdot \rho }$$

(2) $$G\left(S\right)=G\left(X\right)\cdot 2\frac{p_{0}}{q_{0}}$$

where $M_{c}$ is the number average molecular weight between two successive crosslinks (kg/mol), c is the polymer concentration in irradiated solution (g/L), D is the absorbed dose (Gy) and ρ is the solution density (kg/m^3^). The G(X) and G(S) values calculated for G3 and G4 hydrogels are presented in Table 2.

The calculated G(X) values for G3 hydrogels ranged between 0.17–0.23 µmol/J and increased with the absorbed dose, at the same time were considerably higher than G(S) for all radiation conditions. For G4 hydrogels, systematically higher values are obtained for all samples concerning G3, and the proportion between cross-linking and degradation yield was found to be almost equal, G(X) ≈ G(S). This has been attributed to the fact that cross-linking and chain scission processes compete with each other and the final reaction will continue from cross-linking to degradation.

The maximum value of G(X) was found to be 1.22 µmol/J and increased with absorbed dose. In the same trend, also G(S) has increased with absorbed dose, however, their values were lower than correspondent G(X). The G(X) and G(S) values presented in Table 2 are close with other results related to the degradation of chitosan via γ-irradiation, where G(S) = 1.67 µmol/J [41] and irradiation of chitosan in solid state (0.6 µmol/J) or aqueous solution (0.19 µmol/J) [42].

### 2.3. Swelling Degree and Degradation Testing

The outstanding property of hydrogels for wound dressings is their water holding capacity assuring at the same time a moist wound environment beneficial to the healing. Since the blood contains about 90% water, and the excellent swelling properties of the hydrogels make the gel to absorb a large amount of water from the blood and stop bleeding [17]. The absorption property of the hydrogels is also influenced by the nature of the polymers from which the hydrogel is prepared, such as chitosan, which swells readily in biological fluids.

**Figure 2:**
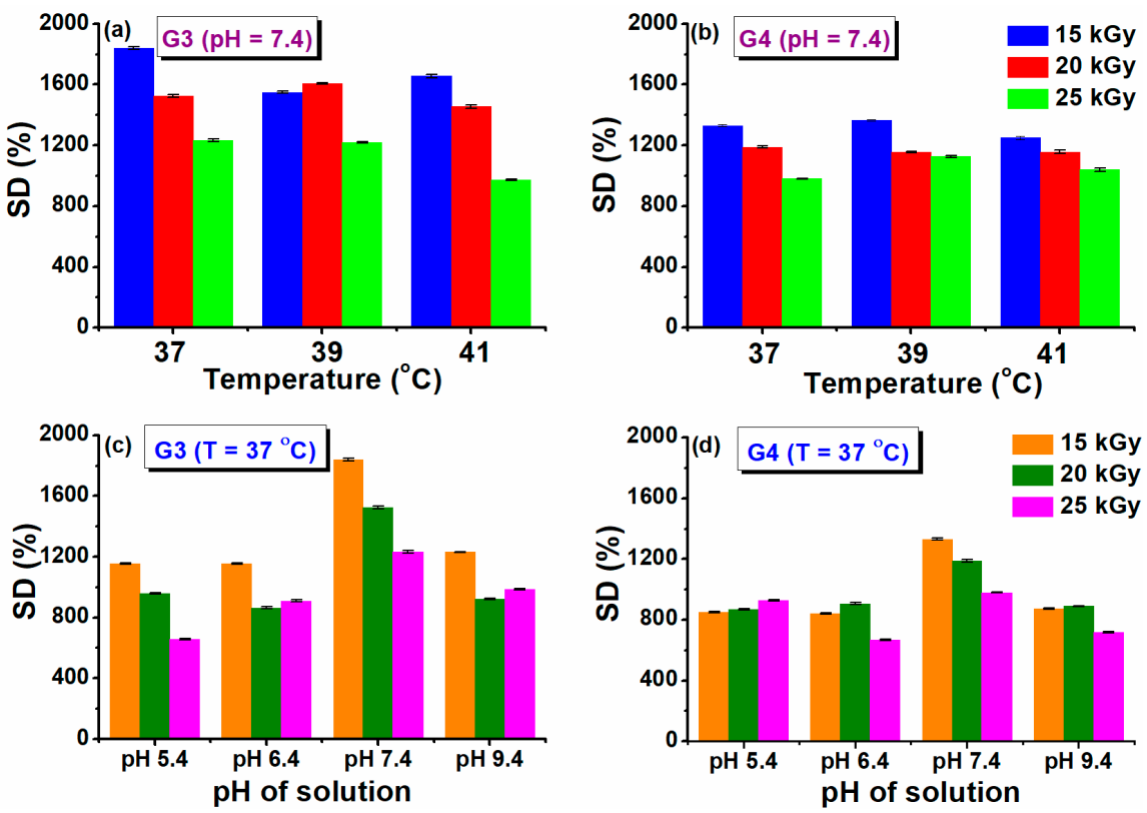
Swelling degree at different temperatures (**a**,**b**) and pH solutions (**c**,**d**).

The maximum swelling capacity of G3 and G4 hydrogels in phosphate buffer saline (PBS, pH = 7.4) and different temperature conditions, similar to normal body temperature (37 °C) and in conditions of body hyperthermia (39–41) °C is shown in Figure 2a,b.

A fast swelling of at least 500% in the first 3 h is observed for all the prepared hydrogels, pointing out towards their porous structure, while at later stages slower modification of swelling is noticed, until the maximum capacity is attained (see also Supplementary Materials Figure S1). The swelling degree (SD%) reached a value above 1800% (PBS, 37 °C) for the G3 hydrogel obtained with 15 kGy. A lower value of 1300% was obtained for G4 hydrogel. For the formulation of G3 hydrogel we used PEG with lower molecular weight, in the case of G4 hydrogels, we used PEO with higher molecular weight, which most probably produces an increase in the viscosity of the polymeric system, but also an increase in the cross-linking density [43]. This behavior is also supported by the results presented in the network studies, where it was shown that the cross-linking density was considerably higher. Similar trends were found for hydrogels based on collagen/PVP/PAA/PEO [44].

Regarding the evolution of SD% with absorbed dose and temperature, this parameter decreased as absorbed dose increased. The reduction in swelling ability is caused by the formation of a cross-linked structure more tightly folded upon irradiation with a higher dose. When the degree of cross-linking increases significantly, the macromolecular network of the hydrogel becomes denser, which prevents the diffusion of the solvent molecules from the swelling medium. When the temperature of the swelling has increased in simulated hyperthermia conditions, a reduction of the SD% was observed by a percentage of 10% at 41 °C.

Healthy and healing skin has a slightly acidic pH (5.5–6.5), while in contrast, the infected chronic wounds have a pH ranging between 7.2 and 9 due to the alkaline secondary products that appeared from the proliferation of bacterial colonies [45]. Therefore, it is important to test the swelling behavior of G3 and G4 hydrogels as a function of pH (5.4–9.4). The results obtained at 37 °C are presented in Figure 2c,d. In this study, it was observed that the G3 and G4 hydrogels exhibited almost the same SD% value at the pH = 5.4, 6.4, and 9.4, having a maximum swelling of 1200%, respectively 850% for the hydrogels prepared at 15 kGy (see also Supplementary Materials, Figure S2 for the time evolution of swelling at various pH). For both types of hydrogels, it was observed a higher SD% of 1800 % (G3), respectively 1300% (G4) when the swelling media had pH = 7.4. Since the hydrogels contain both NH_2_ and COOH moieties derived from CS, NMBA, and acrylic and lactic acids used for CS solubilization, these functional groups can be found in a protonated/deprotonated state. It is well established that hydrogels with a high concentration of charged ionic groups will swell considerably due to osmosis and charge repulsions [46]. When pH > 9, the screening effect of Na+ contained in the swelling environment hinders the SD% of hydrogels [47].

**Figure 3:**
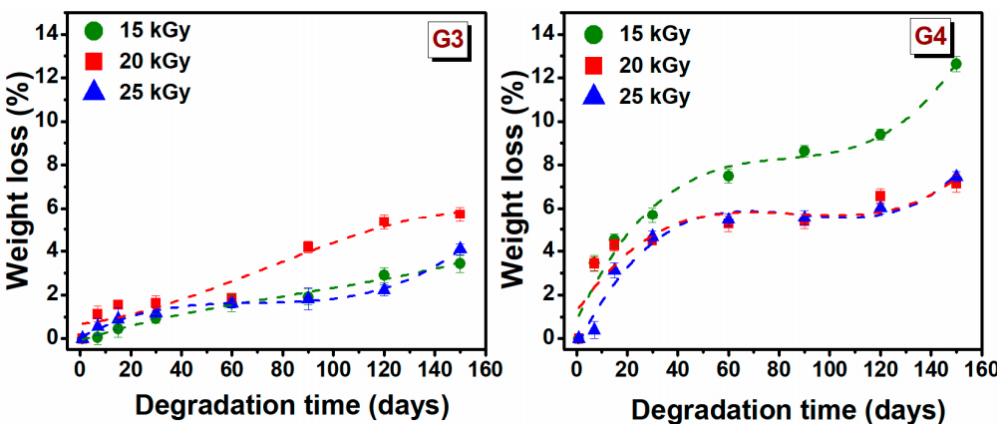
Gradual weight loss (%) of G3 and G4 hydrogels in PBS at 37 °C.

The degradation testing of hydrogels, during incubation for 150 days, based on the weight loss of the material, was monitored in PBS (pH = 7.4) at 37 °C. Degradation testing can help determine the efficiency of cross-linking and the stability of hydrogel. The weight loss (%) of hydrogels depends on the initial concentration of polymer solution, molecular weight, crosslinking agents and swelling properties [48]. The weight loss of G3 and G4 hydrogels at a specific time point is shown in Figure 3.

The weight loss for G3 hydrogels is significantly lower than that of the G4 hydrogels. In the first 30 days, the degradation percentage was 1.2 ± 0.4% for G3 hydrogels, respectively 5 ± 0.6% for the G4 hydrogel. The low rate of degradation of G3 hydrogel can be associated with the permanent crosslinked network formed upon e-beam irradiation as rheological experiments have shown, while a higher degree of degradation of G4 can be correlated with a lower crosslinked hydrogel network. For G3 hydrogel, the free −OH and −NH_2_ groups of the chitosan backbone, are the principal reactive site where the cross-linking take place, so at the end of reaction such groups are no longer available for other interactions. Taking this into account in the degradation process the interaction ability with water will be reduced.

After 150 days of study, it is observed that weight loss gradually increased over time, hydrogels had only lost 5.7% of their initial weight for G3 hydrogel and 12.6% for G4 hydrogel. These results indicated that the hydrogels exhibited a stable structure. However, the trend indicates that at a given moment, the hydrogels will disintegrate almost completely.

### 2.4. Hydrogel Crosslinked Network

Some important network parameters used to assess the cross-linked network structure of hydrogel have been determined. They include polymer volume fraction in the swollen state ($V_{2s}$), the average molecular weight between two successive cross-links ($M_{c}$), cross-link density ($V_{e}$) and mesh size (ξ). Cross-link density ($V_{e}$) is one of the most significant structural parameters of a hydrogel and establishes the weights of fluids that can be retained in its structure. Using the G’ values determined from rheological experiments and based on the rubber elasticity theory, $M_{c}$ can be determined using the following equation [14].

(3) $$M_{C}=\frac{A\rho \mathrm{RT}{\left(V_{2r}\right)}^{2/3}{\left(V_{2s}\right)}^{1/3}}{G\text{’}}$$

where *R* is universal gas constant (8.314 m^3^ Pa/mol K), *T* is the absolute experimental temperature (298.15 °K); *V*_2*r*_ is the polymer volume fraction after e-beam cross-linking, *V*_2*s*_ is the polymer volume fraction of the crosslinked hydrogel in swollen state, ρ is the density of the polymer and the factor *A* equals 1 for an affine network. The effective crosslink density, *V_e_*, of a crosslinked structure can be obtained from the results of rheological measurements using Equation (4):

(4) $$V_{e}=\frac{\rho }{M_{C}}$$

The polymer volume fractions (*V*_2*r*_ and *V*_2*s*_) were calculated as:

(5) $$V_{2r\left(s\right)}=\frac{{\left[1+\left(w_{2r\left(s\right)}-1\right)\cdot \rho_{\mathrm{hydrogel}}\right]}^{-1}}{\rho_{\mathrm{solvent}}}$$

where ρ_hydrogel_ and ρ_solvent_ are the densities of hydrogel and solvent (kg/m^3^), w_2r(s)_ is the weight of the hydrogel after e-beam crosslinking, respectively after swelling (g). The weight ratio of hydrogels after crosslinking (w_2r_) was calculated as: w_2r_ = hydrogel mass after irradiation/hydrogel dry mass. The weight ratio of hydrogels after swelling (w_2s_) was calculated as: w_2s_ = hydrogel mass after swelling/hydrogel dry mass.

The mesh size (ξ) shows the linear distance between consecutive cross-linking points and the space available between the macromolecular chains and was estimated using the following equation [49]:

(6) ξ=ν2s−1/3·Cn2MCMr−12·l

where $C_{n}$ is the Flory characteristic ratio determined as average weight of the contained polymers (PVP = 12.3 [50]; CS = 32.8 [51], AA = 6.7 [52], PEG = 4.0 [53]). $M_{r}$ is the molecular weight of the monomer unit, taken as a weighted average of the molecular weights of PVP = 111.14 g/mol [50], CS = 161.2 g/mol [54], PEG = 44.05 [55], and AA = 72.06 g/mol [52], l is the carbon–carbon bond length (0.154 nm). The experimental values of *G’*, $M_{c}$, $V_{e}$ and *ξ* are summarized in Table 3.

As shown in Table 3, the $M_{c}$ and ξ decreases with increasing of absorbed dose showing a more compact and cross-linked network. The G4 hydrogel crosslinked with 25 kGy, showed a $M_{c}$ parameters larger, in contrast with the hydrogel G3 obtained at the same dose, which means the reduction of cross-linking points. The $M_{c}$ for the investigated hydrogels comprises 2.4–64.7 × 10^3^ kg/mol and 14.9–86.6 × 10^3^ kg/mol, respectively. The results show that the G4 hydrogels prepared with LA and PEO show larger $M_{c}$ and ξ values at 15 kGy and 25 kGy, which suggests that at this dose points, either the crosslinking reaction is not completed, or at doses higher than 20 kGy, the hydrogel network is affected by degradation processes.

Therefore, large values of $M_{c}$ and ξ parameters, reflect a lower cross-linking density. The values of the mesh size are in the range 11.5–67.7 nm, respectively 23.8–59.6 nm, obviously depending on the hydrogels composition and absorbed dose. For G3 and G4 hydrogels, the cross-link density was found to be 1.6–4.2 × 10^−2^ mol/m^3^, respectively 1.2–6.7 × 10^−2^ mol/m^3^ and increased with absorbed dose, only for G4 hydrogel irradiated at a dose of 25 kGy, the $V_{e}$ decreased suggesting network degradation. The above results are very well supported by the experimental data obtained in the degradation study and XPS analysis.

### 2.5. Retention Capability

Being an important factor of the wound dressing, moisture retention capability of hydrogels was investigated and is shown in Table 4. Results indicated high moisture retention capability for both hydrogels compositions. At 2 h, there were no significant differences in moisture retention capacity between the hydrogels, with a range of 96.7%–97.6%. It can be seen in Table 5 that G3 and G4 hydrogels prepared e-beam radiation cross-linking with 15–25 kGy, retain after 24 h between 80.59% and 82% of humidity. Maintaining increased humidity in wounds coated with such a hydrogel ensures a suitable environment for faster healing.

### 2.6. Water Vapor Transmission Rate (WVTR)

In clinical situations, hydrogels are usually used as a primary dressing, attached to the wound area, and then covered by a secondary dressing. The most difficult problem in treating a burned person was the fact that the victim may have lost most of its body liquid due to evaporation and exudation. The WVTR of normal skin is 204 g m^−2^ day^−1^, whereas for injured skin it varies a lot from 279 g m^−2^ day^−1^ to 5138 g m^−2^ day^−1^ depending upon the type of wound [56]. Based on Table 5, it can be seen that the WVTR values of hydrogels are around 167–273 g m^−2^day^−1^. When the irradiation dose increased, the WVTR increased. The hydrogels which were irradiated at 25 kGy have the highest WVTR (272.67 and 245.94 g m^−2^ day^−1^), presenting values of WVTR higher than that of normal skin. Such values cause a faster drying of the wound. On the other hand, the G3 hydrogel, irradiated at 15 kGy has the lowest WVTR (167 g m^−2^ day^−1^). These values of WVTR are beneficial to provide intermittent conditions for a faster wound healing process.

### 2.7. ATR-FTIR

**Figure 4:**
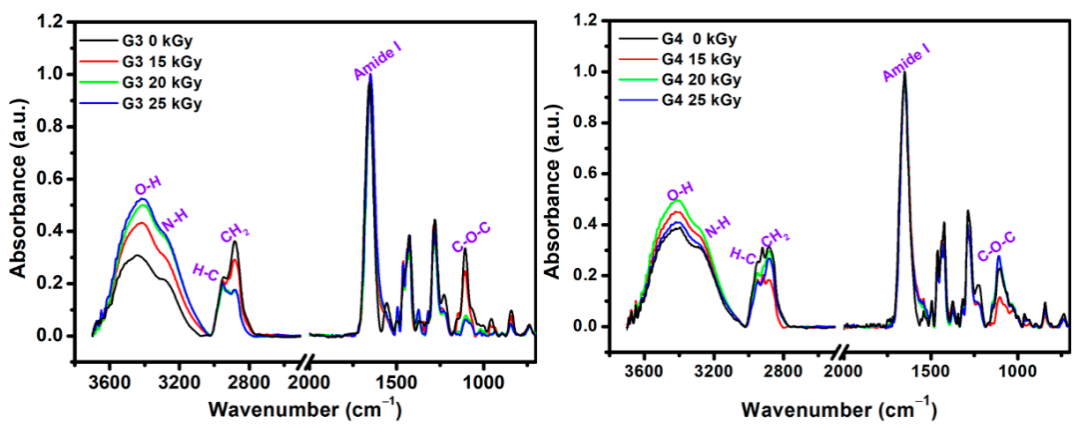
FTIR spectra of lyophilized G3 and G4 hydrogels.

The structural changes that appeared after e-beam irradiation in the hydrogels were investigated by ATR–FTIR. Characteristic FTIR spectra of unirradiated and irradiated G3 and G4 hydrogels are shown in Figure 4, normalized to the highest intensity peak at 1654 cm^−1^.

As expected, the FTIR spectra of the obtained materials contain features originating from each of their component. The main FTIR characteristic absorption bands corresponding to chitosan are: 3356 cm^−1^ and 3293 cm^−1^ (O-H stretch overlapped with N–H stretching), 2800–2900 cm^−1^ (C-H stretch), 1647 cm^−1^ (amide I band, C-O stretch of an acetyl group), 1573 cm^−1^ (amide II band and N-H stretch), 1375 cm^−1^ (amide III, asymmetric C-H bending of CH_2_ group),1149 cm^−1^ (antisymmetric elongation of the C-O-C bridge), and 1062 cm^−1^ indicate skeletal vibration involving the bridge C-O stretch of glucosamine residue [57].

The specific absorption bands corresponding to PVP are: 3470 cm^−1^ (O-H stretching); 2870–2950 cm^−1^(C-H and CH_2_ stretching); 1651 cm^−1^ (C = O and C-N stretching vibration); 1370/1420/1459/1490 cm^−1^ (C-H and CH_2_ deformation vibrations of pyrrolic ring); and 1265/1280 cm^−1^ (stretching vibrations of amide III band) [58]. The main FTIR peaks of PEO are: 2875 cm^−1^ stretching vibration of CH_2_ groups; 1094 cm^−1^ band is derived from the association of CH_2_ groups with the etheric ones of the C-O-C group; 1464 cm^−1^/1342 cm^−1^ deformation vibrations outside the plane (wagging) of CH_2_ groups; 954 cm^−1^ and 840 cm^−1^ rocking plane vibrations of the CH_2_ groups [59].

In the FTIR spectra of the G3 (0 kGy) pre-hydrogel, the main peaks were found at 3440 cm^−1^, assigned to the N-H groups from PVP molecule; 3290 cm^−1^ assigned to the overlapped N-H and O-H groups of chitosan. The peaks in the 2880–2945 cm^−1^ range show stretching vibration of C-H and CH_2_ bonds common of PVP, chitosan and PEO. The band observed at 1654 cm^−1^ caused by the intermolecular inter-polymers H bonds of O-H, N-H of chitosan and C = O of PVP. The band from 1556 cm^−1^ is specific to the amide II of the chitosan molecule. At 1427 cm^−1^ are present the deformation vibrations of CH_2_ bond common of PVP ring. The peak at 1280 cm^−1^ corresponds to the amide III of the PVP molecule and the peak situated at 1105 cm^−1^ corresponding both to the C-O-C and C-O group of chitosan and PEO. In the FTIR spectra of the G3 cross-linked hydrogel, within 3700–3000 cm^−1^ range the increase of bands intensity and the shift to lower wavenumbers up to 3407 cm^−1^ as function of absorbed dose was observed. Moreover, the intensity of C-H and CH_2_ groups has decreased after e-beam cross-linking. The stretching vibration peak at of 1654 cm^−1^ is getting broader, while the contribution of amide II peak in chitosan appears just as a shoulder, suggesting a strong participation of O-H and N-H groups in the chemical reaction under e-beam irradiation. On the other hand, the decrease in the intensity of the C-O-C peak from 1105 cm^−1^ could be correlated with the shortening of polymeric chain length as a consequence of a higher absorbed dose. The above observation is also supported by XPS investigations.

The unirradiated (0 kGy) G4 pre-hydrogel showed a wide absorption band from 3600 to 2800 cm^−1^. In this region, the main peaks were identified at 3394 and 3284 cm^−1^ (O-H and N-H from chitosan and PVP), 2922 cm^−1^, and 2882 cm^−1^ (C-H and CH_2_ bonds common of both chitosan, PEO and PVP).

In the range, 1700–650 cm^−1^ the peaks corresponding to both chitosan and PVP were found: 1650 cm^−1^ (amide I band), 1430 cm^−1^ (C = O, and C-N), 1280 cm^−1^ (amide III), 1105 cm^−1^ (C-O-C).

For G4 hydrogel cross-linked with 20 kGy and 25 kGy, it was observed the loss of peak from 2920 cm^−1^ associated with C-H bond, most probably due to high cross-linking evidenced for this composition. Other significant changes in the FTIR spectra of G4 hydrogels were observed for the peaks situated around 1105 cm^−1^, whose intensities increased with the irradiation dose, suggesting a more significant contribution of some oxidative processes due to the higher absorbed dose.

### 2.8. X-Ray Photoelectron Spectroscopy (XPS)

**Figure 5:**
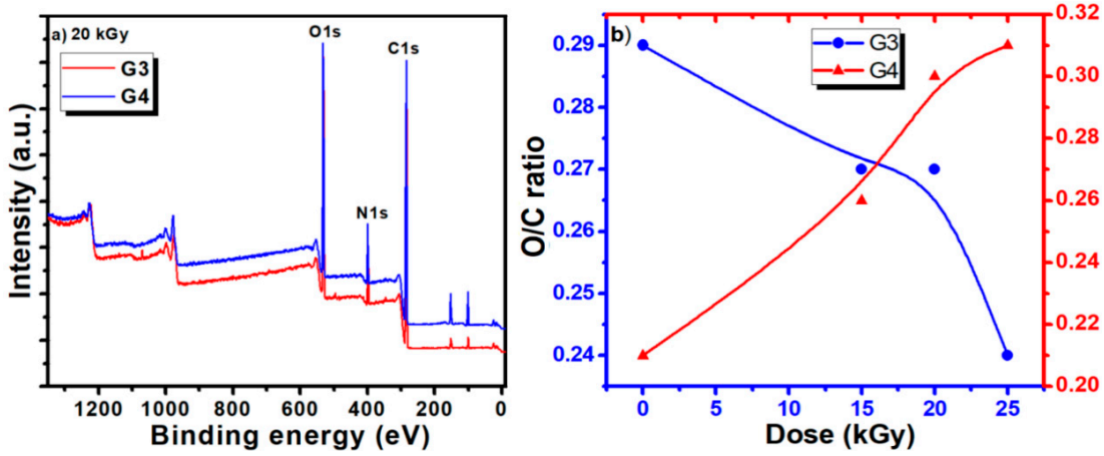
(**a**) Comparative survey spectra for G3 and G4 hydrogels recorded for 20 kGy absorbed dose; (**b**) dependence of O/C ratio as function of the hydrogel type (G3 and G4) and irradiation dose.

Typical survey spectra for the G3 and G4 hydrogels are presented in Figure 5a. They present a similar aspect, revealing the presence of carbon, nitrogen, and oxygen. The elemental composition of the unirradiated CS-PVP-PEG/PEO polymeric mixtures and G3, G4 hydrogels synthesized by e-beam irradiation at various doses, as determined from the XPS survey spectra, are reported in Table 6, evidencing only small variation of the composition. 

The calculated ratios between oxygen and carbon concentration (O/C ratio) and nitrogen and carbon concentration (N/C ratio) are also included.

The evolution of the O/C ratios for the G3 and G4 hydrogels is illustrated in Figure 5b. For the G3 hydrogel based on CS-PVP-PEG, the O/C ratio shows just a slight decrease for irradiation doses below 20 kGy, suggesting the partial preservation of the pre-hydrogel initial chemical structure, while upon higher doses a more pronounced decrease is noticed, related to cross-linking of the material.

For the G4 hydrogel based on CS-PVP-PEO, the O/C ratio presents an ascendant trend with the irradiation dose, suggesting an easy opening of epoxide ring in acid in the case of PEO conducting to more pronounced oxidation state is in this case. N/C ratio present an increasing trend for both hydrogels, for irradiation dose up to 20 kGy, while at 25 kGy a sharp decrease is noticed for the G4 gel, pointing again towards a different behavior for this experimental condition, as previously evidenced by the gel fraction and radio-chemical yield of degradation investigations.

**Figure 6:**
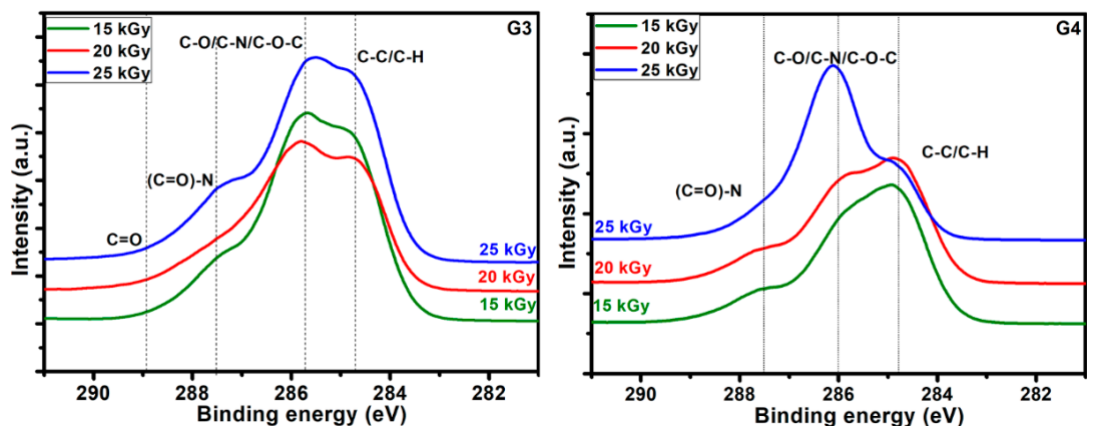
High resolution spectra in the C1s region for the G3 hydrogels and respectively G4 hydrogels.

The C1s high resolution spectra recorded for the hydrogels obtained at various absorbed doses, for both G3 and G4, are displayed in Figure 6. They evidence on one hand the specificity given by the different composition of the initial polymeric solution which undergoes the electron beam irradiation procedure, and on the other hand the modifications induced in the chemical bonding as function of the irradiation dose.

**Figure 7:**
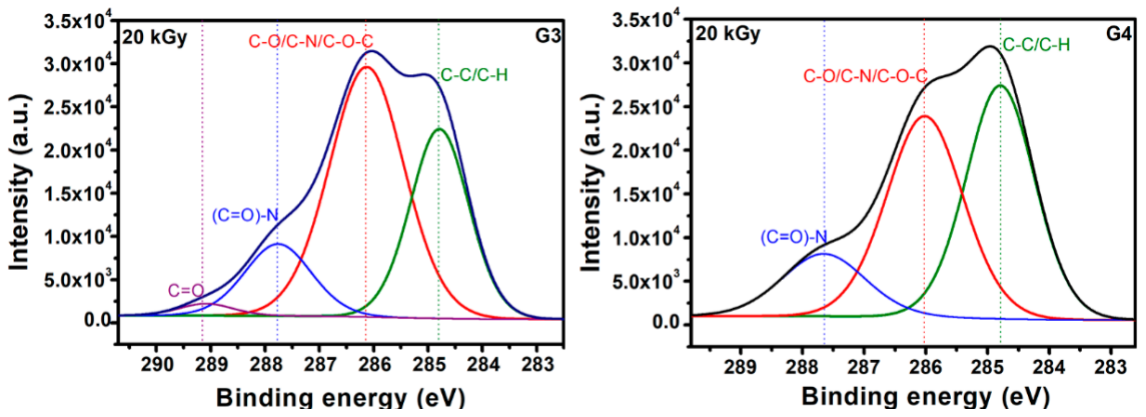
Chemical bonds as evidenced upon deconvolution of C1s high resolution spectra for G3 hydrogels and respectively G4 hydrogels.

Typical deconvolutions performed to reveal various bonds present in the materials, for both G3 and G4 hydrogels are presented in Figure 7. The fitting was considered completed when the chi values minimized, below 1. In the case of G3 hydrogels, the deconvolution of C1s spectrum was considering four components, as follows: C-C/C-H at 285 eV, C-O/C-N/C-O-C at 286 eV, (C = O)-N bonds at 288 eV and C = O bonds at 289 eV. For the G4 hydrogel, the deconvolution was performed using three components, as follows: C-C/C-H bonds at 285 eV, C-O/C-N/C-O-C bonds at 286 eV and (C = O)-N bonds at 288 eV [60,61].

**Figure 8:**
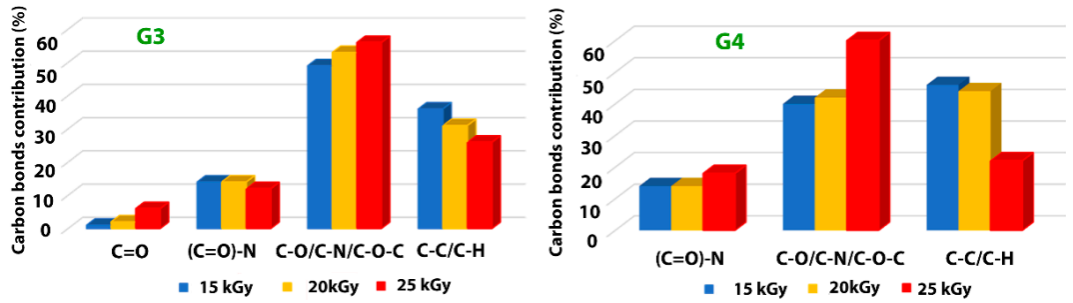
Dependence of the various carbon-related bonds on the irradiation dose for G3 hydrogels and respectively G4 hydrogels.

In Table 7 are presented the contributions of various carbon bonds as function of irradiation dose for G3 and G4 hydrogels, while in Figure 8 are represented their dependencies upon the irradiation dose.

For both investigated hydrogels, the amount of C-C/C-H bonds decreases with irradiation dose suggesting a breaking of the polymeric chains due to e-beam irradiation. This is accompanied by the increase of the contribution of C-O/C-N/C-O-C-bonds with the absorbed dose, which points out towards a cross-linking of the broken chains to the surrounding oxygen or nitrogen atoms. For the G3 hydrogels, the amount of carbon bonded in C = O bond is also increasing with irradiation dose, while the contribution of (C = O)-N reveals slight decreasing which is in accordance with the N/C ratio trend. In the case of G4 hydrogel, one can notice the drastic decrease of C-C/C-H bonds at large irradiation dose of 25 kGy with an increase of the contribution counted for C-O/C-N/C-O-C-bonds, as well as that of (C = O)-N bonds, suggesting more accentuated oxidative degradation in this case.

The results indicate, once again, following specific hydrogel investigations revealed in paragraph 2.1 and FTIR analysis, that the chemical structure of the G4 hydrogel based on CS-PVP-PEO is significantly degraded under irradiation dose of 25 kGy.

### 2.9. Evaluation of Encapsulation Efficiency, Drug Loading Capacity, and In Vitro Drug Release Study

**Figure 9:**
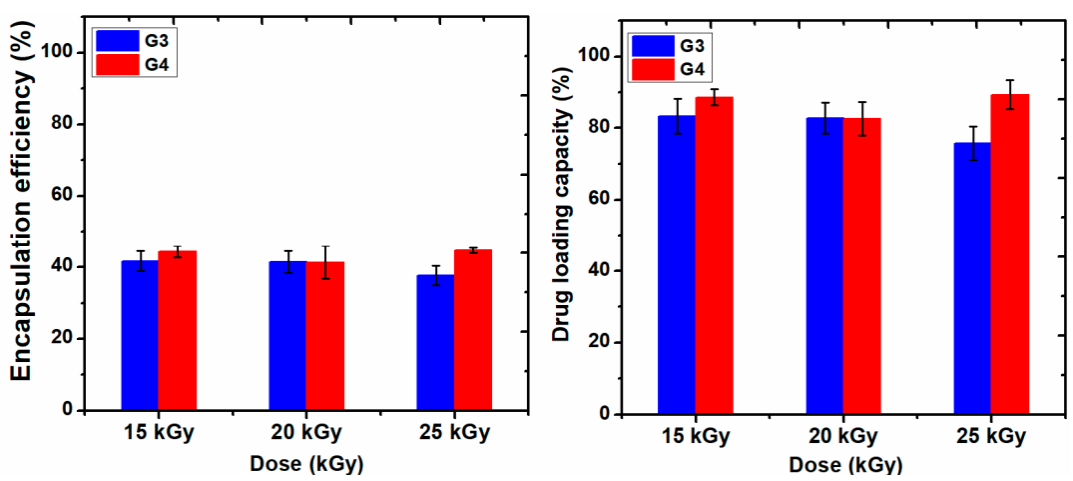
The encapsulation efficiency (%) and drug loading capacity (%) of hydrogels.

**Figure 10:**
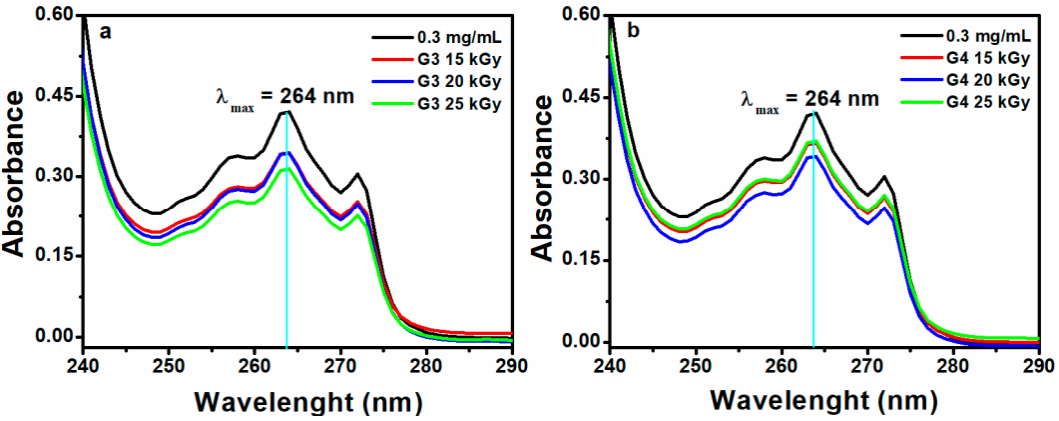
UV-Vis absorption spectra of IBU in (**a**) G3 and (**b**) G4 hydrogels (0.3 mg/mL–UV-Vis spectra for the control sample without hydrogel).

Figure 9 shows the data of encapsulation efficiency (EE) and drug loading capacity (LC) of ibuprofen in the G3 and G4 hydrogels in correlation with absorbed dose. Figure 10a,b show typical UV-Vis absorption spectra of IBU in G3 and G4 hydrogels. The corresponding calibration curve of IBU solubilized in EtOH within 0.05–0.6 mg/mL and UV-Vis absorption spectra are presented in the Supplementary Materials, Figure S3a,b.

Ibuprofen is stabilized in the hydrogel matrix through H bonding or polar interactions between drug molecules with the ionized groups of the polymer chain and other hydrophobic interaction between the hydrophobic moieties of the drug and hydrogel. Since the release of a drug from hydrogel is essentially a mesh-controlled diffusion, this process will be influenced by mesh size and cross-link density of the hydrogels as well as by the size of the released molecules. As can be seen in Figure 9, the EE (%) of IBU decreased with the absorbed dose for G3 hydrogels. A decrease of 5% was observed for the hydrogel cross-linked with a dose of 25 kGy, most likely due to the increase of cross-linking degree, thus the active substance is effectively prevented from entering throughout the macromolecular network of the hydrogel. The typical UV-Vis absorption spectra of IBU in G3 and G4 hydrogels are presented in Figure 10a,b.

The drug loading capacity of IBU was reduced below 80% in the case of G3 hydrogels when the absorbed dose is 25 kGy, on the contrary, the drug loading capacity in the G4 hydrogels increased at the dose of 25 kGy, probably due to the breaking of polymer chains due to e-beam irradiation.

**Figure 11:**
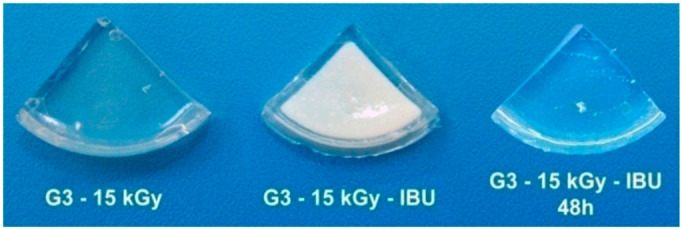
Picture of hydrogels obtained upon irradiation at 15 kGy, after encapsulation with ibuprofen (IBU) and respectively after complete drug release.

In Figure 11 it is presented the encapsulation process of IBU in G3 hydrogels and the appearance of G3 hydrogel loaded with IBU and a piece of hydrogel after IBU complete releasing. The release profile of the IBU was afterwards investigated at pH = 7.4 and 9.4, corresponding to non-infected and infected wounds, respectively.

**Figure 12:**
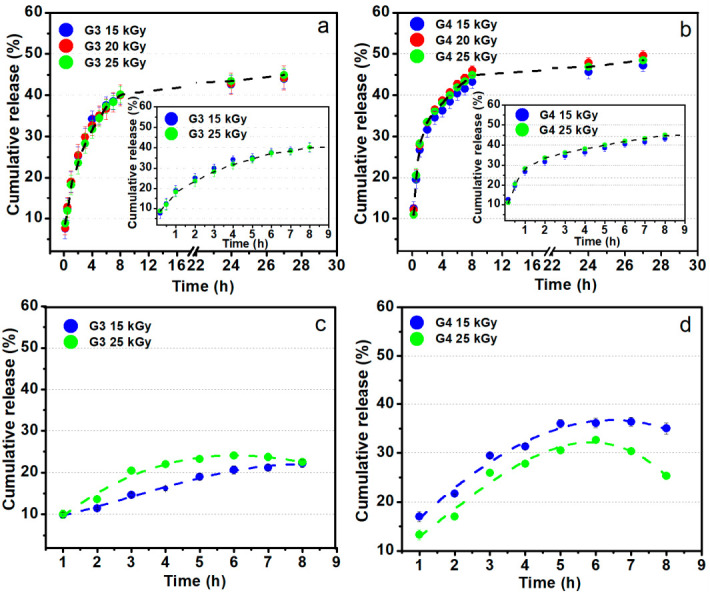
Ibuprofen release profiles from G3 and G4 hydrogels in PBS pH 7.4 (**a**,**b**) and sodium carbonate–sodium bicarbonate buffer pH 9.4 at 37 °C (**c**,**d**).

Figure 12a,b shows the release profiles of the ibuprofen loaded in G3 and G4 hydrogels up to 27 h in PBS (pH = 7.4, 37 °C), pointing out similar release behavior regardless of absorbed dose. An inset showing the release behavior in the first 8 h is also included. One can notice that about 30–45% of ibuprofen is released in the first 5 h for both types of compositions, equivalent to around 30 mg. This behavior points out that the drug release rate from the IBU loaded hydrogel is abrupt at the starting time, afterwards, the absorbed IBU is still released from hydrogel up to 30 h. Prolonged-release of IBU from a hydrogel-type polymeric matrix may be beneficial if it is considered that the normal half-life is 1–3 h, thus can help to maintain an optimum concentration in the body [62]. A faster release rate of IBU during the first 3 h with a linear dependence was observed for both hydrogels composition. At the starting time, the release process is controlled by the swelling capacity of the hydrogel while as the polymer networks become more hydrated, the release of IBU takes place through a diffusion process. This behavior was pointed out by other studies based on the evaluation of IBU release from different hydrogel matrix having in composition chitosan or other formulation typically designed for topical administration of ibuprofen [63]. Djekic et al. showed that the maximum amount of IBU that can be released from a reference gel, namely Nurofen^®^ gel was approximately about 50% after 6 h [64]. Comparing with the above-mentioned study, our hydrogels can release within 40–50% of IBU after 8 h. More than that, our data are very close to that of other hydrogels formulation with similar composition [65].

Figure 12c,d shows the ibuprofen loaded in G3 and G4 hydrogels behavior regarding the release profiles for up to 8 h in sodium carbonate–sodium bicarbonate buffer (pH = 9.4, 37 °C). These data simulate the drug release on the site of an infected wound. In contrast with the experiments at pH 7.4, in the weak alkaline media, the IBU release is lower, reaching 25–35% in the first 5 h. Such behavior could be the result of lower swelling degree at pH 9.4 as previously presented in the swelling experiments (Figure 2).

## 3. Materials and Methods

### 3.1. Materials

Chitosan (CS, degree of N-deacetylation = 85% M_w_ = 1.9–3.1 × 10^5^ g/mol, poly(vinylpyrrolidone) (PVP, M_w_ = 3.6 × 10^6^ g/mol), poly(ethylene glycol) (PEG, M_w_ = 2 × 10^4^ g/mol), poly(ethylene oxide (PEO, M_w_ = 3 × 10^5^ g/mol), N’N-methylene-bis(acrylamide) (NMBA, assay 99%, M_w_ = 154.17 g/mol), sodium hydroxide (NaOH, 99% assay, reagent ACS), acrylic acid (AA, anhydrous 99%, M_w_ = 72.06 g/mol and lactic acid (LA, 90% assay, reagent ACS, M_w_ = 90.08 g/mol) were purchased from Sigma Aldrich (St. Louis, MO, USA). Ibuprofen (IBU) was provided by a local pharmaceutical company. Deionized (DI) water was prepared in our laboratory. All reagents were commercially available and used without further purification.

### 3.2. Electron Beam Synthesis of Hydrogels

**Figure 13:**
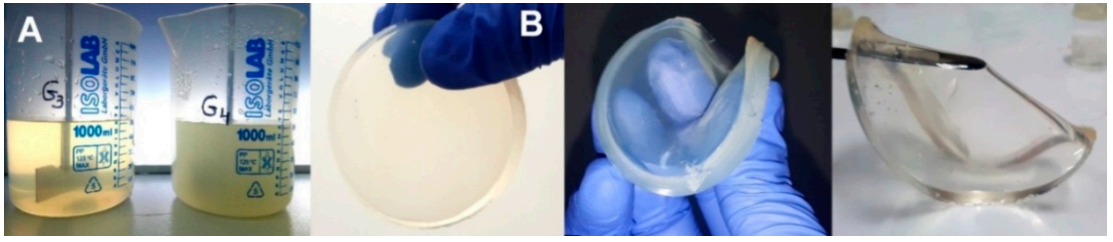
(**a**) Pre-hydrogel solutions before e-beam cross-linking; (**b**) digital photographs of the cross-linked hydrogels.

CS (0.5%, *w*/*v*) was dissolved in an aqueous solution of acrylic acid (AA, 0.35% *w*/*v*) or lactic acid (LA, 0.45%*w*/*v*). Before the addition of CS, the pH of the two solutions was checked with a pH-meter (AA: pH = 2.7; LA: pH = 2.5). CS was solubilized in an ultrasonic bath (50 °C, 2.5 h, 37 kHz, 130 W). After complete solubilization of CS, various amounts of PVP (7%, *w*/*v*), PEG (1.2%, *w*/*v*), PEO (1.2%, *w*/*v*), and NMBA (0.5%, *w*/*v*) were added and the mixture was stirred at room temperature until complete homogenization. The final pH of the polymer solutions was adjusted to 5.5 with NaOH (1 M). The composition of the hydrogels is shown in Table 8. To reduce the oxidative degradation effects of the polymeric materials during irradiation, the homogenized pre-hydrogel solutions were then poured into test tubes and were centrifuged at 500 rpm (5 min) to remove the air introduced during mechanical stirring, as shown in Figure 13a. Thereafter, pre-hydrogel solutions were poured into plastic Petri dishes (32 mm diameter), each pre-hydrogel sample having a mass of 3 ± 0.1 g and a thickness of 5 mm. Before irradiation, the Petri dishes containing the unirradiated pre-hydrogel solution were vacuum packed. E-beam irradiation of the pre-hydrogel samples was performed with a 6 MeV linear electron accelerator (ALID 7) owned by the National Institute for Laser, Plasma and Radiation Physics (INFLPR), Măgurele-Romania.

Irradiation was carried out at an average beam current of 10 μA, a pulse length of 3.75 μs, a pulse repetition rate of 50 Hz and a filament voltage of 12 V [66]. The absorbed doses applied to the samples were 15, 20, 25 kGy, and the average dose rate was 3 kGy/min. The dosimetry was performed using a graphite calorimeter [67]. The typical aspect of the obtained hydrogels, presented in Figure 13b, reveals that the samples are transparent, flexible and with good resistance to mechanical stress. The chemical structures of the polymeric substrate and the proposed CS-PVP-PEG-PAA hydrogel network after e-beam cross-linking are shown in Scheme 1.

### 3.3. Characterization of Hydrogels

#### 3.3.1. Determination of Gel Fraction

The hydrogels were cut into small pieces and vacuum-dried at 40 °C until a constant weight was achieved, then immersed in DI water for 48 h at constant room temperature. After 48 h, the samples were taken out and dried to constant weight. The gel fraction (G) and sol fractions (s) were calculated using the following equations:

(7) $$G\left(\%\right)=\left(\frac{W_{d}}{W_{0}}\right)$$

(8) s=1−G

where $W_{0}$ and $W_{d}$ were the weights (g) of dried hydrogels before and after water extraction. All measurements were carried out at room temperature (25 °C) in triplicate for each sample and all values were expressed as mean value and standard deviation of three individual samples.

#### 3.3.2. Sol-Gel Analysis

To determine the formation of insoluble gel and evaluation of e-beam radiation cross-linking process, the sol-gel analysis was performed. Following this step, the main parameters of radiation cross-linking process as: $D_{g}$—gelation dose (the lowest dose needed to initiate macroscopic gel formation); $p_{0}{/q}_{0}$—the ratio of radiation yield of scission ($p_{0}$) to radiation yield of cross-linking ($q_{0})$ were evaluated using the GelSol95 software (developed by Division of Applied Radiation Chemistry, Lodz University of Technology, Poland).This software is used for the numerical analysis of gel/absorbed dose curves based on the Charlesby–Rosiak equation [68]:

(9) $$s+\sqrt{s}=\frac{p_{0}}{q_{0}}+\left(2-\frac{p_{0}}{q_{0}}\right)\left(\frac{D_{v}+D_{g}}{D_{v}+D}\right),$$

where s is the sol fraction of polymer (mass fraction of the dissolved material), $p_{0}$ is the chain scission yield (average number of main chain scissions per monomer unit and per unit dose), $q_{0}$ is the cross-linking yield (proportion of monomer units cross-linked per unit dose), D is the absorbed dose (kGy) and $D_{v}$ is the virtual dose (kGy). $D_{v}$ represents the necessary dose to transform the actual sample into a sample of the most probable molecular weight distribution of Mw/Mn = 2 [69].

For compositions which form insoluble fractions upon radiation cross-linking, the ${\;p}_{0}{/q}_{0}$ ratio should be less than 2, this value confirming that the cross-linking processes predominated [35].

#### 3.3.3. Swelling Degree and Degradation Testing

The dried hydrogels of known weights (~100 mg) were left to swell in phosphate buffer solution (PBS, pH = 7.4, 37–41 °C) and buffer solutions (citrate, bicarbonate) of different pH values (5.4, 6.4, and 9.4) at 37 °C. Subsequently, the samples were removed from the buffer solution at subsequent time intervals in the range 0.3–50 h, blotted with paper, weighed, and immersed again. The degree of swelling was determined by the following equation:

(10) $$\mathrm{SD}=\frac{\left(W_{s}-W_{0}\right)}{W_{0}}\cdot 100$$

where $W_{s}$ is the weight (g) of the sample after swelling and${\;W}_{0}$ is the initial weight of the dry sample.

Degradation experiments were performed in PBS (pH = 7.4) at 37 °C. After certain time intervals (7, 15, 30, 60, 90, 120, and 150 days), the swollen hydrogels were taken out from the solution, blotted with paper, weighed and then PBS was refreshed. The weight loss (%) at each time interval was calculated using Equation (11), where $W_{i}$ is the initial weight of sample and $W_{f}$ is the final weight of each sample.

(11) $$\mathrm{Weight}\;\mathrm{loss}\;\left(\%\right)=\frac{\left(W_{i}-W_{f}\right)}{W_{f}}\cdot 100$$

All measurements were carried out in triplicate for each sample and all values were expressed as mean value and standard deviation of three independent samples.

#### 3.3.4. Moisture Retention Capability

To calculate the moisture retention capability (MRC), each hydrogel pad was cut into 1 × 1 cm^2^ pieces, put individually in Petri dishes and the initial weight ($W_{0}$) at room temperature (25 °C) was measured. At different time intervals (2–24 h), the Petri dishes left in ambient conditions were weighed ($W_{t}$). MRC was determined as the water loss rate and the ratio of water holding in the hydrogel, which was calculated with Equation (12) [70]:

(12) $$\mathrm{MRC}=\frac{W_{t}}{W_{0}}\cdot 100\%.$$

#### 3.3.5. Water Vapor Transmission Rate

The measurement of water vapor transmission rate (WVTR) of the hydrogel was performed according to the monograph of the European Pharmacopoeia [71]. The moisture permeability of the hydrogel was determined from the weight loss of a cylindrical bottle having an internal diameter of 15 mm containing 10 mL of DI water. The bottle was capped on the mouth with a hydrogel disc (20 mm diameter), sealed with parafilm, and kept in an oven at 37 °C for 24 h. After 24 h, the bottle was withdrawn from the oven and weighed again. The WVTR was determined as follows:

(13) $$\mathrm{WVTR}=\frac{\left(W_{0}-W_{t}\right)}{\left(A\times 24\right)}\cdot 10^{6}{g\;m}^{-2}{\;\mathrm{day}}^{-1},$$

where A is the area of the bottle mouth (mm^2^), $W_{0}$ and $W_{t}$ are the weight (g) of the bottle before and after placing it in the oven, respectively.

#### 3.3.6. Fourier Transform Infrared (FT-IR) Analysis

The FT-IR spectra of unirradiated and irradiated samples were taken with a Spectrum 100 instrument (Perkin Elmer, USA). The samples were lyophilized prior FT-IR analysis. The FT-IR spectra were acquired in ATR mode in the (4000–650) cm^−1^ range with 50 scans/sample and a resolution of 4 cm^−1^.

#### 3.3.7. X-Ray Photoelectron Spectroscopy (XPS)

Investigations regarding the surface chemical composition of the CS-PVP-PEG-PAA/PEO materials resulted upon e-beam irradiation were completed by using the X-ray Photoelectron Spectroscopy (XPS) method. The measurements were performed on dried samples (upon lyophilization) by using a K-Alpha Thermo Scientific (ESCALAB™ XI+, East Grinstead, UK) spectrometer equipped with a 180° double-focusing hemispherical analyzer. Peaks’ positions were calibrated according to the standard C1s peak (284.6 eV). Survey spectra were recorded at pass energy of 50 eV to determine the elemental composition of the CS/PVP materials, while high-resolution spectra for C1s, O1s, and N1s binding energy regions were measured at pass energy of 20 eV to evaluate the elemental bonding states of the as-resulted materials. The data processing was completed by using advanced Avantage data software (Thermo Avantage v5 9921).

#### 3.3.8. Evaluation of Encapsulation Efficiency, Drug Loading Capacity and In Vitro Drug Release Study

The ibuprofen (IBU), encapsulation efficiency (EE), and drug loading capacity (LC) of the CS-PVP-PEG-PAA hydrogels were determined spectrophotometrically with a UV/Vis Spectrophotometer (Varian Cary 100 Inc, USA). For this experiment, 2000 mg of IBU was dissolved in 100 mL of ethanol (EtOH). From this solution, 10 mL EtOH + IBU (W_1_ = 200 mg) was taken, and then dried hydrogel sample (W = 100 + 0.2 mg) was added.

The hydrogel sample vials, including EtOH + IBU solution, were sealed and then stirred (6 h, 50 rpm) using a water bath shaker at a temperature of 37 ± 0.1 °C. The remaining solution (W_2_) was filtered, and the precipitate (drug-loaded hydrogel) was washed with 2 mL of EtOH to remove the drug absorbed on the surface of the hydrogel. The IBU hydrogels were dried in a vacuum oven at 37 °C until constant weight.

The concentration of IBU in the filtrate EtOH solution (W_2_) was determined by measuring the absorbance value at 264 nm, corresponding to the maximum absorption peak of IBU, and using a predetermined calibration curve based on Beer–Lambert law (Figure S3).The EE is defined as the percentage of the drug-loaded in a polymeric matrix and the LC is the amount of total drug loaded divided by the total polymeric matrix weight. The percentage of EE and LC of hydrogels were calculated using the equations below [72]:

(14) $$\mathrm{EE}=\frac{\left(W_{1}-W_{2}\right)}{W_{1}}\cdot 100$$

(15) $$\mathrm{LC}=\frac{\left(W_{1}-W_{2}\right)}{W}\cdot 100$$

The residual concentration of IBU was calculated, taking into account the dilution factor and the volume of the solution. The drug release of IBU from the hydrogel was studied by adding the drug-loaded hydrogel to a beaker containing 100 mL PBS at pH 7.4. The solution was continuously stirred at 37 ± 0.1 °C. At time intervals in the range, 0–27 h, 5 mL liquid samples were removed from the beaker to allow measuring the drug concentration, while 5 mL fresh PBS solution was added replacing the removed buffer.

The released IBU amount was determined by UV-Vis absorption considering in this case the peak positioned at the wavelength of 221 nm. The absorption curve and calibration curve of IBU in PBS are presented in the Supplementary Materials, Figure S4. The cumulative release of IBU (%) was determined according to the following formula:

(16) $$\mathrm{Cumulative}\;\mathrm{release}\;\mathrm{of}\;\mathrm{IBU}\left(\%\right)=\frac{C\cdot V_{t}+V_{r}\sum_{i=0}^{n-1}C_{i}}{\mathrm{Weight}\;\mathrm{of}\;\mathrm{hydrogel}\cdot \mathrm{LC}\left(\%\right)}\cdot 100\%.$$

C is the sample concentration at time t_n_, Vt is the total volume of release medium (100 mL, PBS), Vr is the removed volume (5 mL) and the Ci is the initial sample concentration at time t_i_. The experiments were performed in triplicate.

## 4. Conclusions

In this study, chitosan-poly(vinyl-pyrrolidone)-poly (ethylene glycol)/poly (ethylene oxide)-poly(acrylic)/poly(lactic) acid hydrogels were successfully synthesized by one step e-beam cross-linking to be used for rapid healing and pain release of infected skin wounds. Investigations of chemical composition by FTIR and XPS confirmed that hydrogels were successfully prepared, with deterioration with respect to the optimum hydrogel properties only for irradiation doses above 20 kGy.

The hydrogels showed high gel fractions (96%) even at lower absorbed dose. The swelling capacity decreased with the absorbed dose, the hydrogels reaching equilibrium after 8 h of immersion in PBS at various pH and temperatures. The hydrogels showed good stability, lower rate of degradation and superabsorbent properties in simulated hyperthermia conditions (37–41 °C) and different pH intervals. The shape of the hydrogels was not affected even after 48 h in the above conditions, as well as no evidence of hydrogel dissolution in studied media.

The network parameters ($M_{C}$, $V_{e}$ and ξ) indicated the formation of a cross-linked structure with a nanostructured mesh with typical dimension in the range 11–67 nm. The WVTR values ranged from 167.21 and 272.67 g m^−2^ day^−1^ confirming that all hydrogels can be used to control body fluid loss and maintain a moist environment for infected skin wounds. This behavior is accompanied by the favorable maintenance of humidity for more than 6 h and considering that this time represents the normal frequency of changing a dressing, ensure a favorable environment for wound healing.

Moreover, the synthesized hydrogels have a significant loading capacity for IBU, in the range of 75–89% depending on the absorbed dose, ensuring the possibility of incorporating a therapeutic dose needed for severe pains. The IBU was released from the macromolecular network of the hydrogels in a proportion below 30% in the first 2 h, reaching a maximum after 8 h, demonstrating the controlled release capacity, both for pHs of 7.4 and 9.4, corresponding to the case of non-infected and infected wounds, respectively. The results point out that the optimum irradiation doses for obtaining hydrogels with the best biomedical properties are between 15–20 kGy.

Further research in order to evidence the antimicrobial response of the hydrogels for various strains, including Gram-negative and Gram-positive bacteria and yeasts are considered. The investigations will be continued as well with the evaluation of viability, cell proliferation, and migration for various cell lines of fibroblasts and keratinocytes. This information will allow the determination of the optimum formulation of the hydrogels dressings based on chitosan-poly(vinyl-pyrrolidone)-poly (ethylene glycol)/poly (ethylene oxide)-poly(acrylic)/poly (lactic) acid for wound healing applications.

## Figures and Tables

**Table 1:** Degradation vs. cross-linking ratio, gelation dose and virtual dose of hydrogels calculated according to the Charlesby–Rosiak equation.

Hydrogel	$p_{0}{/q}_{0}$	$D_{g}\;(\mathrm{kGy})$	$D_{v}(\mathrm{kGy})$
G3	0.28	0.10	2.53
G4	0.51	0.12	−0.73

**Table 2:** Radiation chemical yields of crosslinking and scission of G3 and G4 hydrogels.

Dose (kGy)	Sample Code	Radiation Chemical Yields (μmol/J)	
		G(X)	G(S)
15	G3	0.17	0.09
	G4	1.10	1.12
20	G3	0.20	0.11
	G4	1.08	1.11
25	G3	0.23	0.13
	G4	1.22	1.25

**Table 3:** Network parameters of the G3 and G4 hydrogels.

**G3**				
**Dose(kGy)**	**G’ (Pa)**	**M_c_ (kg/mol)**	**V_e_ (mol/m^3^)**	**ξ (nm)**
15	2.3	64.7	1.6	67.7
20	3.0	49.3	2.1	55.9
25	70.0	2.4	4.2	11.5
**G4**				
**Dose(kGy)**	**G’ (Pa)**	**M_c_ (kg/mol)**	**V_e_ (mol/m^3^)**	**ξ (nm)**
15	2.3	86.6	1.2	59.6
20	13.6	14.9	6.7	23.8
25	4.2	51.5	1.9	42.2

**Table 4:** Moisture retention capability at different time intervals of G3 and G4 hydrogels.

Hydrogel	Dose (kGy)	MRC (%)			
		t = 2 h	t = 2 h	t = 2 h	t = 2 h
G3	15	97.31	94.94	92.59	82.00
	20	96.88	94.49	92.40	81.85
	25	96.88	94.49	92.40	81.85
G4	15	97.59	95.19	93.13	81.98
	20	97.65	94.91	92.61	81.48
	25	96.73	94.23	92.12	80.59

**Table 5:** Water vapor transmission rate of hydrogels.

Hydrogel	Dose (kGy)		
	15	20	25
G3-WVTR (g m^−2^ day^−1^)	167.21	228.50	272.67
G4-WVTR (g m^−2^ day^−1^)	209.96	208.36	245.94

**Table 6:** Elemental composition for unirradiated and irradiated G3 and G4 hydrogels as function of absorbed dose.

Material/Element	C (%)	O (%)	N (%)	O/C	N/C
Unirradiated G3	73.84 ± 0.10	21.50 ± 0.11	4.67 ± 0.23	0.29	0.06
G3					
15 kGy	72.12 ± 0.11	19.76 ± 0.11	8.12 ± 0.15	0.27	0.11
20 kGy	72.82 ± 0.11	19.99 ± 0.11	7.19 ± 0.15	0.27	0.10
25 kGy	73.20 ± 0.10	17.36 ± 0.12	9.44 ± 0.13	0.24	0.13
Unirradiated G4	78.34 ± 0.10	16.60 ± 0.12	5.06 ± 0.22	0.21	0.06
G4					
15 kGy	73.81 ± 0.10	19.03 ± 0.11	7.16 ± 0.15	0.26	0.10
20 kGy	71.36 ± 0.11	21.14 ± 0.11	7.50 ± 0.15	0.30	0.11
25 kGy	70.64 ± 0.11	25.61 ± 0.10	3.75 ± 0.24	0.36	0.05

**Table 7:** Distribution of chemical bonds for both G3 and G4 irradiated hydrogels, as function of irradiation dose.

Material/Bond Type	C = O	(C = O)-N	C-O/C-N/C-O-C	C-C/C-H
**G3**				
15 kGy	1.00	14.00	49.00	36.00
20 kGy	2.00	14.00	53.00	31.00
25 kGy	6.00	12.00	56.00	26.00
**G4**				
15 kGy	-	14.00	40.00	46.00
20 kGy	-	14.00	42.00	44.00
25 kGy	-	18.00	60.00	22.00

**Table 8:** Samples composition of hydrogels.

Hydrogel Code	Composition (%, *w*/*v*)							
	CS	PVP	PEG	PEO	AA	LA	NMBA	H_2_O
G3	0.5	7	1.20	-	0.35	-	0.5	90.45
G4	0.5	7	-	1.20	-	0.45	0.5	90.35

## Supplementary Materials

Supplementary Materials can be found at https://www.mdpi.com/1422-0067/21/23/9236/s1.

## Abbreviations

AA	Acrylic acid
c	Polymer concentration in irradiated solution
Cn	Flory characteristic ratio
CS	Chitosan
D	Absorbed dose
Dg	Gelation dose
DI	Deionized water
D_v_	Virtual dose
e-beam	Electron beam
EE	Encapsulation efficiency
FTIR	Fourier Transform Infrared
G	Gel fraction
G(S)	Radiation yield of the chain scission
G(X)	Radiation yield of the crosslinking
IBU	Ibuprofen
LA	Lactic acid
LC	Drug loading capacity
Mc	Average molecular weight between two successive crosslinks
Mr	Molecular weight of the monomer unit
MRC	Moisture retention capability
NMBA	N’N-methylene-bis(acrylamide)
p_0_	Chain scission yield
PAA	Poly(acrylic acid)
PAL	Poly(lactic acid)
PBS	Phosphate buffer saline
PEG	Polyethylene glycol
PEO	Polyethylene oxide
PVP	Poly(N-vinyl pyrrolidone)
q_0_	Crosslinking yield
s	Sol fraction
SD	Swelling degree
V2r	Polymer volume fraction after e-beam crosslinking
V2s	Polymer volume fraction in the swollen state
Ve	Crosslink density
WVTR	Water vapor transmission rate
XPS	X-ray photoelectron spectroscopy
ξ	Mesh size
ρ	Density of the solution
