# Supplementary material for: One Step e-Beam Radiation Cross-Linking of Quaternary Hydrogels Dressings Based on Chitosan-Poly(Vinyl-Pyrrolidone)-Poly(Ethylene Glycol)-Poly(Acrylic Acid)
Source: Int J Mol Sci. 2020 Dec 3;21(23):9236. doi: 10.3390/ijms21239236 (PMC7731230; doi:10.3390/ijms21239236)
Supplement: Supplementary file 1 [file ijms-21-09236-s001.pdf]

Supplementary Material

One step e-beam radiation cross-linking of quaternary hydrogels dressings based on Chitosan-Poly(Vinyl-Pyrrolidone)-Poly(ethylene Glycol)-Poly(Acrylic acid)

Ion Călina [1,2], Maria Demeter [1,*], Anca Scărișoreanu [1], Veronica Sătulu [1] and Bogdana Mitu [1,*]

[1] National Institute for Lasers Plasma and Radiation Physics, 409 Atomiștilor, 077125 Măgurele, Romania; calina.cosmin@inflpr.ro (I.C.); anca.scarisoreanu@inflpr.ro (A.S.); veronica.satulu@inflpr.ro (V.S.)

[2] University of Bucharest, Faculty of Physics, 405 Atomiștilor, 077125 Măgurele, Romania

* Correspondence: maria.dumitrascu@inflpr.ro (M.D.); mitub@infim.ro (B.M.)

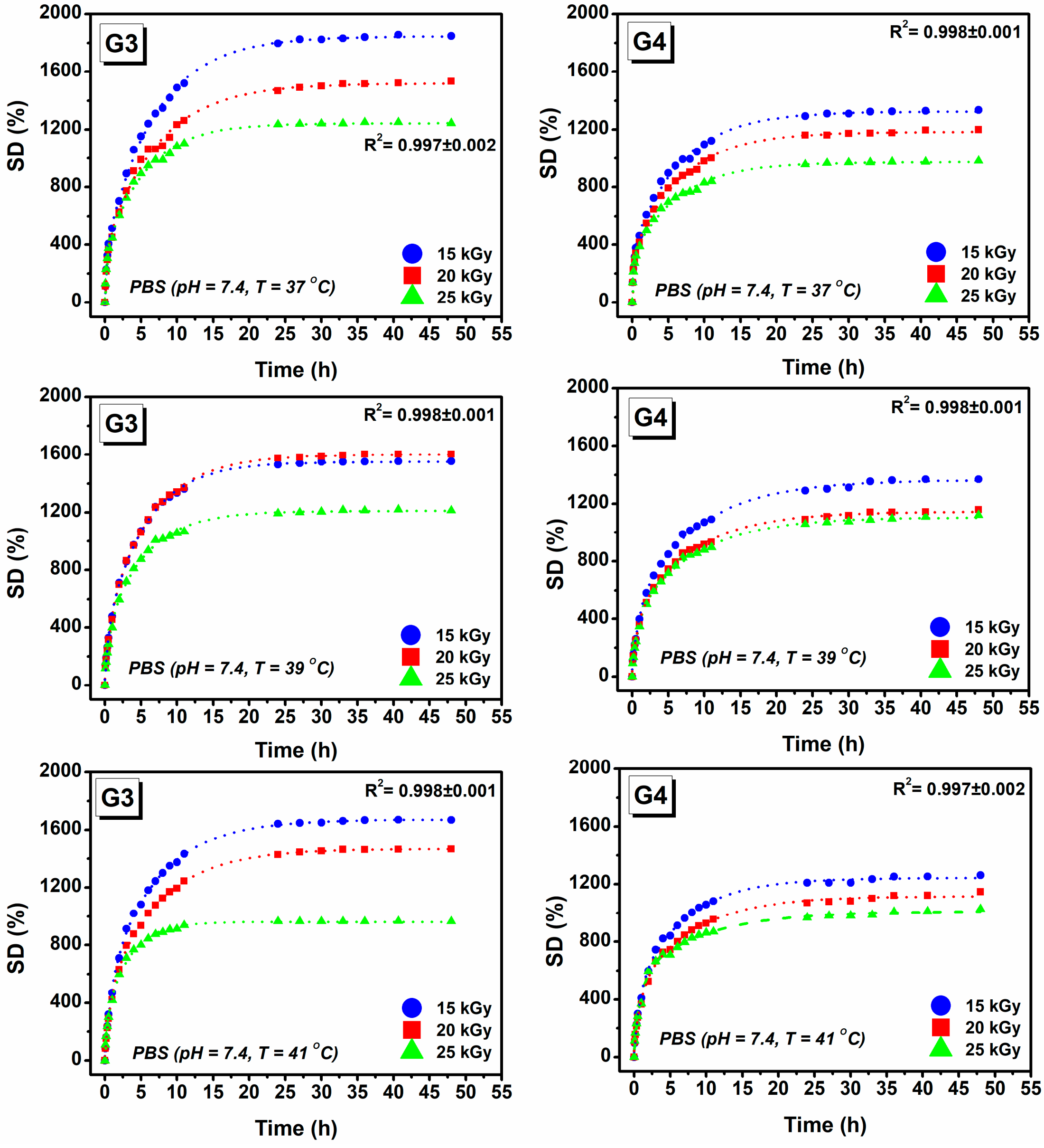

Figure S1. The time evolution of swelling degree of hydrogels in PBS (pH = 7.4) at temperatures in the

range 37–41 °C

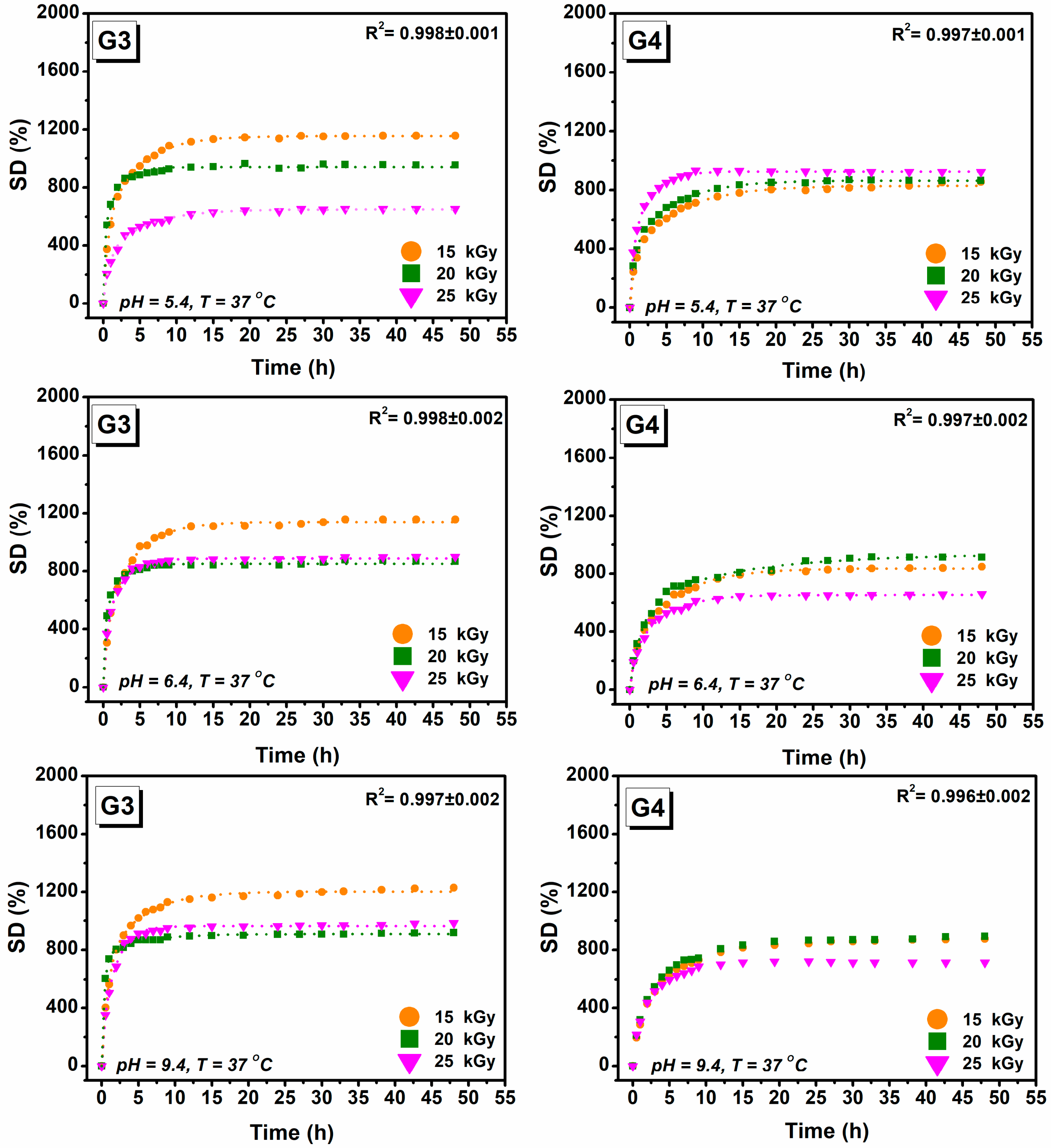

Figure S2. The time evolution of swelling degree of hydrogels in the pH range 5.4–9.4 at 37 °C.

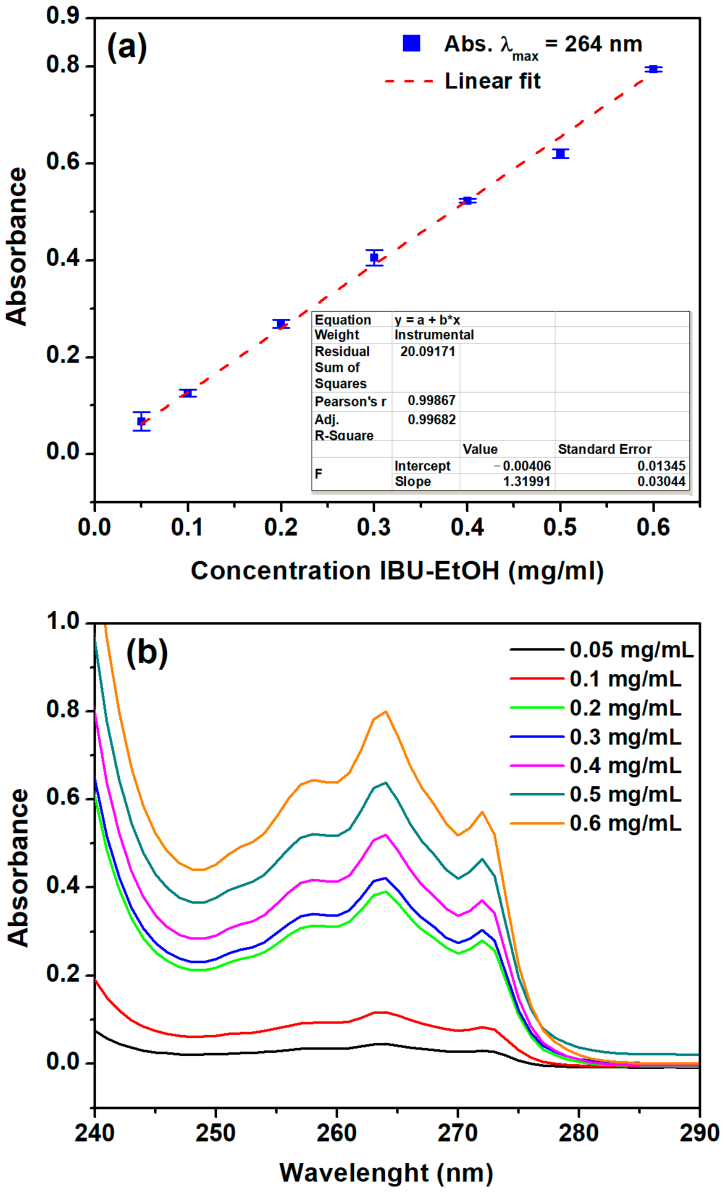

Figure 3. (**a**) Calibration curve of IBU-EtOH (0.05–0.6 mg/mL) at 264 nm; (**b**) UV-Vis absorption spectra of IBU in EtOH.

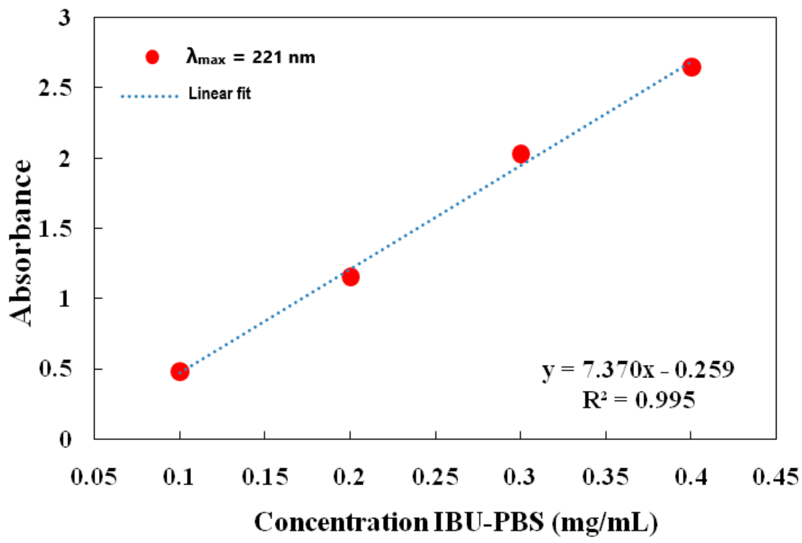

Figure S4. Calibration curve of IBU-PBS (0.1–0.4 mg/mL) at 221 nm
